# 3D-printed boluses for radiotherapy: influence of geometrical and printing parameters on dosimetric characterization and air gap evaluation

**DOI:** 10.1007/s12194-024-00782-1

**Published:** 2024-02-13

**Authors:** Simone Giovanni Gugliandolo, Shabarish Purushothaman Pillai, Shankar Rajendran, Maria Giulia Vincini, Matteo Pepa, Floriana Pansini, Mattia Zaffaroni, Giulia Marvaso, Daniela Alterio, Andrea Vavassori, Stefano Durante, Stefania Volpe, Federica Cattani, Barbara Alicja Jereczek-Fossa, Davide Moscatelli, Bianca Maria Colosimo

**Affiliations:** 1 Department of Mechanical Engineering, Politecnico di Milano, Via La Masa, 1, 20156 Milano, Italy; 2 Department of Chemistry, Materials and Chemical Engineering “Giulio Natta”, Politecnico di Milano, Piazza Leonardo da Vinci, 32, 20133 Milano, Italy; 3 Division of Radiation Oncology, IEO European Institute of Oncology, IRCCS, Milano, Italy; 4 Clinical Department, Bioengineering Unit, National Center for Oncological Hadrontherapy (CNAO), Pavia, Italy; 5 Unit of Medical Physics, IEO European Institute of Oncology, IRCCS, Milano, Italy; 6 Department of Oncology and Hemato-Oncology, University of Milan, Milano, Italy

**Keywords:** Additive manufacturing, 3D printing, Bolus, Radiotherapy

## Abstract

The work investigates the implementation of personalized radiotherapy boluses by means of additive manufacturing technologies. Boluses materials that are currently used need an excessive amount of human intervention which leads to reduced repeatability in terms of dosimetry. Additive manufacturing can solve this problem by eliminating the human factor in the process of fabrication. Planar boluses with fixed geometry and personalized boluses printed starting from a computed tomography scan of a radiotherapy phantom were produced. First, a dosimetric characterization study on planar bolus designs to quantify the effects of print parameters such as infill density and geometry on the radiation beam was made. Secondly, a volumetric quantification of air gap between the bolus and the skin of the patient as well as dosimetric analyses were performed. The optimization process according to the obtained dosimetric and airgap results allowed us to find a combination of parameters to have the 3D-printed bolus performing similarly to that in conventional use. These preliminary results confirm those in the relevant literature, with 3D-printed boluses showing a dosimetric performance similar to conventional boluses with the additional advantage of being perfectly conformed to the patient geometry.

## Introduction

External beam radiation therapy (EBRT) often requires additional medical devices to be successfully delivered, such as boluses, which are routinely utilized to shift the dose build-up region and avoid proximal target under-dosage. Boluses should perfectly conform to the patient's surface, should be easy to put on a daily basis, pleasant, be tissue equivalent in density and in radiation absorbance/attenuation behavior and be able to accommodate for changing patient anatomy over therapy [1, 2].

Boluses that are commercially available can be split into three types: gel-based, moldable, and wax-based boluses. The main disadvantage of these types of commercial boluses is the difficulty to conform them to irregular skin surfaces, such as nose, ear, neck, and scalp, with the possibility of undesired air gaps and the reduction of the reproducibility of the treatment [3, 4].

The requirement to place the bolus material directly on the skin of the patient is what makes this area ideal for an exploration into personalized medicine. With the possibilities provided by additive manufacturing (AM) or three-dimensional (3D) printing, the fabrication of personalized boluses, printed directly using the contour taken from the patient computed tomography (CT) data set, routinely acquired during the radiotherapy planning phase, has been a trend in recent years.

Numerous research and clinical trials have been carried out, as 3D-printed boluses have numerous advantages in terms of patient shape conformity, fast time manufacturing, repeatability, and the amount of details when reproducing patient geometry [5–10].

**Fig. 1:**
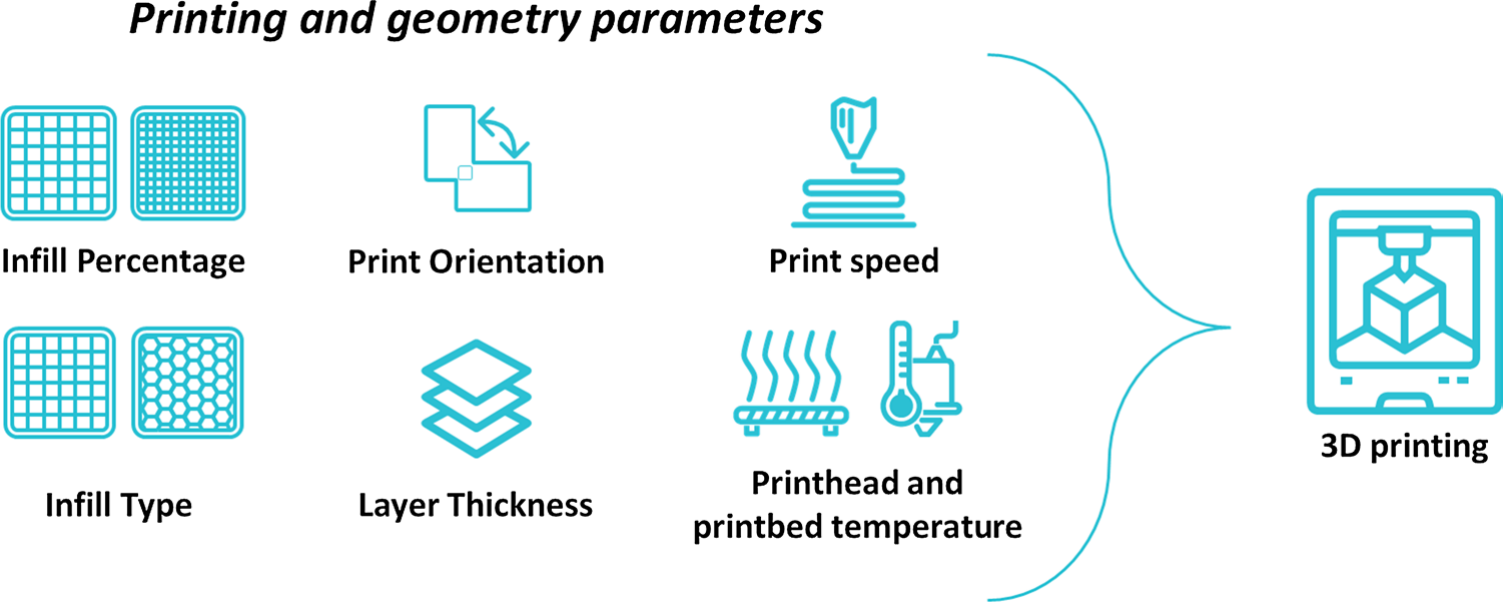
Example of printing and geometry parameters affecting the quality of a printed part

For a 3D-printed bolus, numerous parameters can be changed including process parameters (nozzle size, filament size, printhead temperature, printbed temperature, printing speed) and geometry parameters (layer thickness, infill geometry, infill density, fill angles, width, etc.). In the literature, boluses were printed with infill density variation from 10 to 100%, with rectilinear infill pattern mostly [11]. What is often missing in the literature is a systematic approach aimed at producing customized boluses while taking full advantage of all the degrees of freedom allowed by 3D printing, including the choice of material, shape, and printing as well as the possibility to rapidly test the effectiveness of the proposed solutions (Fig. 1).

This work aims to: (i) determine the effect of infill type and density on the radiation beam behavior to obtain an optimal combination of such parameters, and (ii) test this combination by simulating patient-specific boluses for the nose and the cheek area. Patient-specific boluses will be generated from phantom CT scans, 3D printed at the Politecnico di Milano, evaluated and compared in terms of air gap and shifting of the depth-dose profile, using as standard the traditional boluses used at European Institute of Oncology IRCCS (IEO) in Milan, Italy.

This paper is organized as follows: in materials and methods section, the procedural steps taken to derive the results, from the methodology for fabricating the parallelepipeds for material and infill characterization, to the design of patient-specific boluses are described; in results section, the outcomes of the experiments that were conducted on the different head districts samples are presented; in discussion section, the obtained results on air gap and dosimetry are analyzed, based on prior findings; final conclusions on basis of results and discussions are made.

## Materials and methods

### Optimal printing parameters selection

#### 3D printing technology

The printer used to fabricate the samples was a Sharebot 42 (Sharebot, Italy), a Cartesian 3D printing system with a Bowden extruder, installed at the laboratories of Politecnico di Milano.

For the current work, a general-purpose nozzle with an internal diameter of 0.4 mm was used. Once defined nozzle size and material, the main process parameters, temperature and print speed, were set accordingly to manufacturer indications to obtain the best printability of the selected filament.

#### Tested materials

Two materials were used to print the samples: polylactic acid (PLA) and thermoplastic polyurethane (TPU). PLA, a thermoplastic material derived from corn starch, is resistant to warpage during the print process and does not release any odor. PLA was used in the form of filament and was supplied by Sharebot itself. The hardest variant of TPU filaments, named *FiloFlex Hard*, was chosen and provided by the supplier FiloAlfa (FiloAlfa, Italy). This was done to retain the flexibility characteristics of the material while ensuring a good printability. These materials were chosen because the bolus substance must be odorless, non-sticky, and harmless to the skin [12].

In Table 1, printer and filament specific parameters for both materials are listed.

**Table 1:** Print parameters for PLA and TPU

Parameter	PLA	TPU
Density (kg/m^3^)	1240	1280
Layer height (mm)	0.2	0.3
Loops	3	4
Speed (mm/s)	60	12
Extrusion multiplier	1	1.5
Extruder temperature	250	210
Bed temperature	50	30
Retraction	Yes	No

With regard to the experiments with PLA, which focused on the evaluation of the dose shift and not on the pure geometric conformation, since it is known that the physical properties of the material may change over time depending on storage conditions, two batches of materials were tested.

#### 3D model development

The 3D models chosen to be printed were created on SolidWorks software (Dassault Systèmes SE, Vélizy-Villacoublay, France) and were sliced with Slic3r (free 3D slicing engine software for 3D printers).

A standard iterative design process was followed:

- initial ideation for material and infill characterization with parallelepipeds;
- prototyping of the models for air gap studies;
- final models for dosimetric testing.

Based on the results obtained, on each step, the new iterations led to the modeling of patient-specific boluses.

#### Printing parameters

**Fig. 2:**
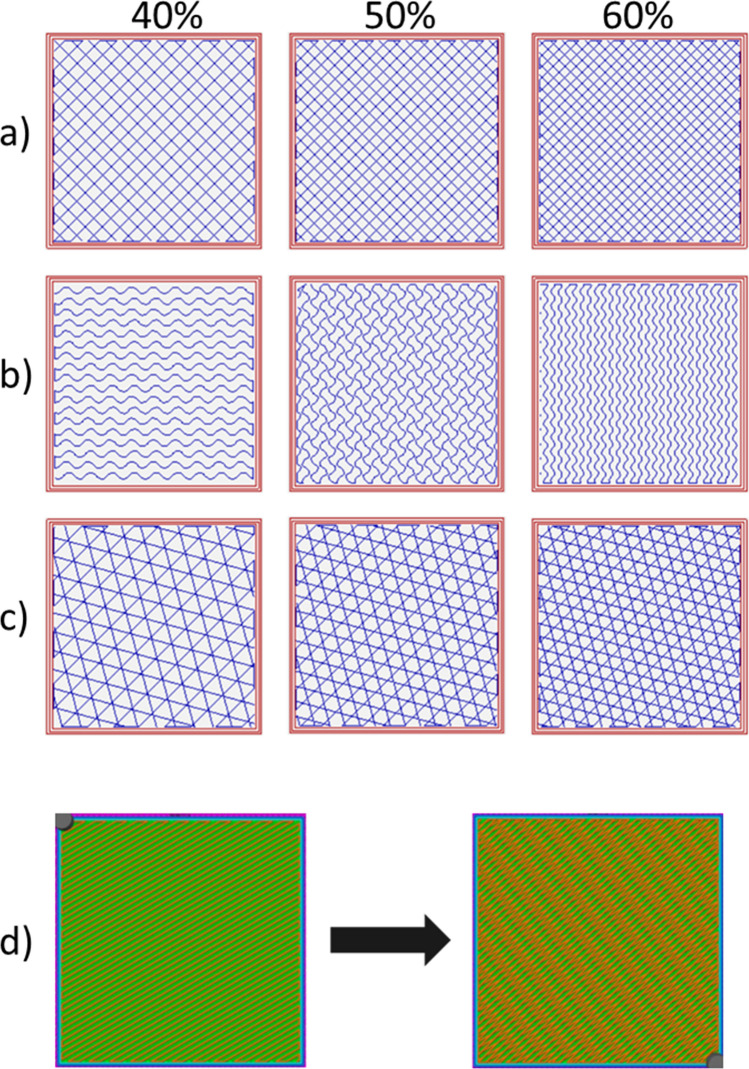
Tested infill: **a** grid, **b** gyroid, **c** cubic, ranging from 40 to 60%, **d** rectilinear infill in subsequent different orientations, example at 50%

Parallelepipeds of size 50 × 50 × 5 mm were modeled, and rectilinear, grid, gyroid, and cubic were chosen as testing geometry infill types (Fig. 2). Two different ways of designing the infill patterns were investigated:

- alter infill density uniformly;
- alter orientations of subsequent infill layers by a constant angle increment.

The first modality of infill variation was implemented in grid, gyroid, and cubic infills. Rectilinear was not taken into consideration for testing this method because it was already tested in one previous work [11], and it is widely studied in the literature for this kind of application. This is why the second modality of infill variation was implemented specifically for the rectilinear infill. Moreover, the second method could not accommodate any other of the chosen infill geometries since gyroid, cubic and 3D honeycomb will not retain the original geometrical characteristics if subsequent layers are offset by a certain angle. To this end, to generate the rotating infill the slicing software Simplify3D (Simplify3D, Ohio, United States) was used. The angular offset between subsequent infill layers was varied from 10° to 20° with an increment of 5°.

For each infill pattern, three different infill densities were chosen: 40%, 50%, and 60%. A duplicate of each condition has been printed, for a total of 36 samples (Table 2).

**Table 2:** Configurations tested

Variation	Pattern	Infill percentage (%)	Orientation angle	Number of samples
Uniform infill	Grid	40	–	2
		50	–	2
		60	–	2
	Gyroid	40	–	2
		50	–	2
		60	–	2
	Cubic	40	–	2
		50	–	2
		60	–	2
Rotational infill	Rectilinear	40	10°	2
			15°	2
			20°	2
		50	10°	2
			15°	2
			20°	2
		60	10°	2
			15°	2
			20°	2
			Total samples	**36**

#### Dose-depth profile evaluation

**Fig. 3:**
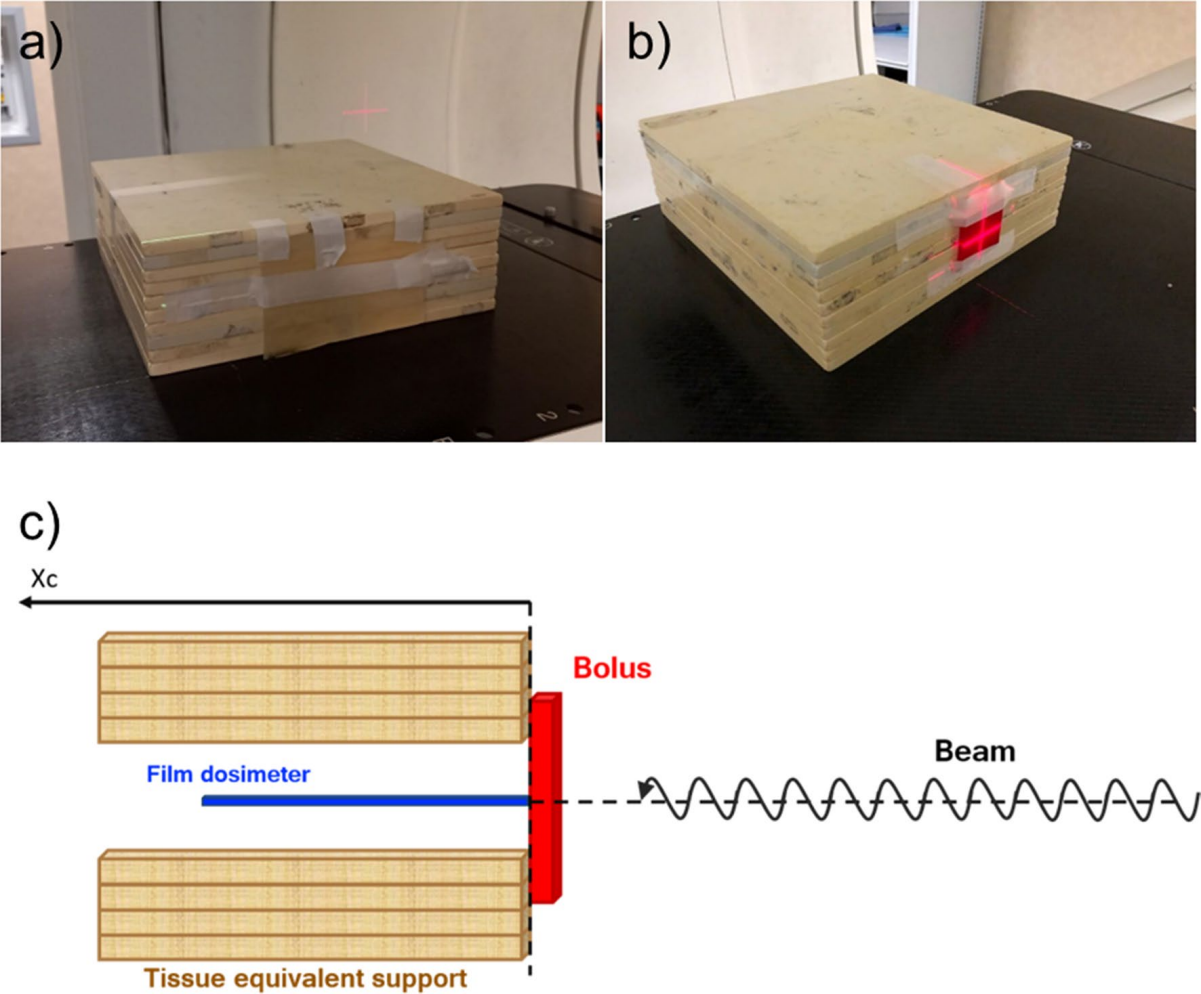
Irradiation set-up: **a** conventional bolus, **b** 3D-printed bolus, and **c** scheme of the configuration

To assess the best printing configuration, the attenuation provided by 3D-printed boluses was evaluated for a 6-MV clinical photon beam on a water-equivalent RW3 slab phantom (Sun Nuclear, Florida, USA) in three configurations at the Division of Radiation Oncology of IEO: without bolus, with a commercial bolus, and with the eight 3D-printed boluses, thus obtaining ten measures. Irradiations were performed with the Vero® System (BrainLAB, Feldkirchen, Germany) delivering 200 monitor units (MU), with a dose rate of 500 MU/min, using a 10 × 10 cm^2^ open field with 90-degree gantry angle at 100 cm skin-to-source distance (SSD). The 3D-printed boluses and the commercial bolus were fixed to the RW3 phantom laterally, as for the treatment plan comparison setting. A Gafchromic EBT3 film (International Specialty Products, Wayne, NJ) was placed between the phantom slabs providing dose profile measurements (Fig. 3). Both calibration and measurement films were scanned 48–72 h after irradiation using a desktop flat-bed transmission Epson Expression Scanner 10,000 XL (Epson, Long Beach, CA). The film scanner was operated with a resolution of 72 dpi in the 48-bit red–green–blue (RGB) mode. The red color channel was extracted a posteriori from the images to maximize readout sensitivity; film analyses were performed using the Film QA Pro software (Ashland Inc., Covington, USA).

Boluses performances were evaluated in terms of the shift of depth-dose profile, comparing them with the traditional boluses. The same workflow was employed for dosimetric characterization in 2.2.2.

### Patient-specific boluses

#### Nose boluses and air gap characterization

Infill parameters from the previous optimization process (2.1) were chosen to test bolus samples printed directly from computed tomography (CT) scan of a phantom, thus simulating a patient’s skin surface. The DICOM files from the CT scan were processed with Slicer3D, a 3D reconstruction and segmentation software.

This was done by inserting a threshold filter which will segregate the sections in the image based on the intensity values of voxels. The filter keeps within the segment the regions whose intensity values fall within the limits of the threshold.

**Fig. 4.**
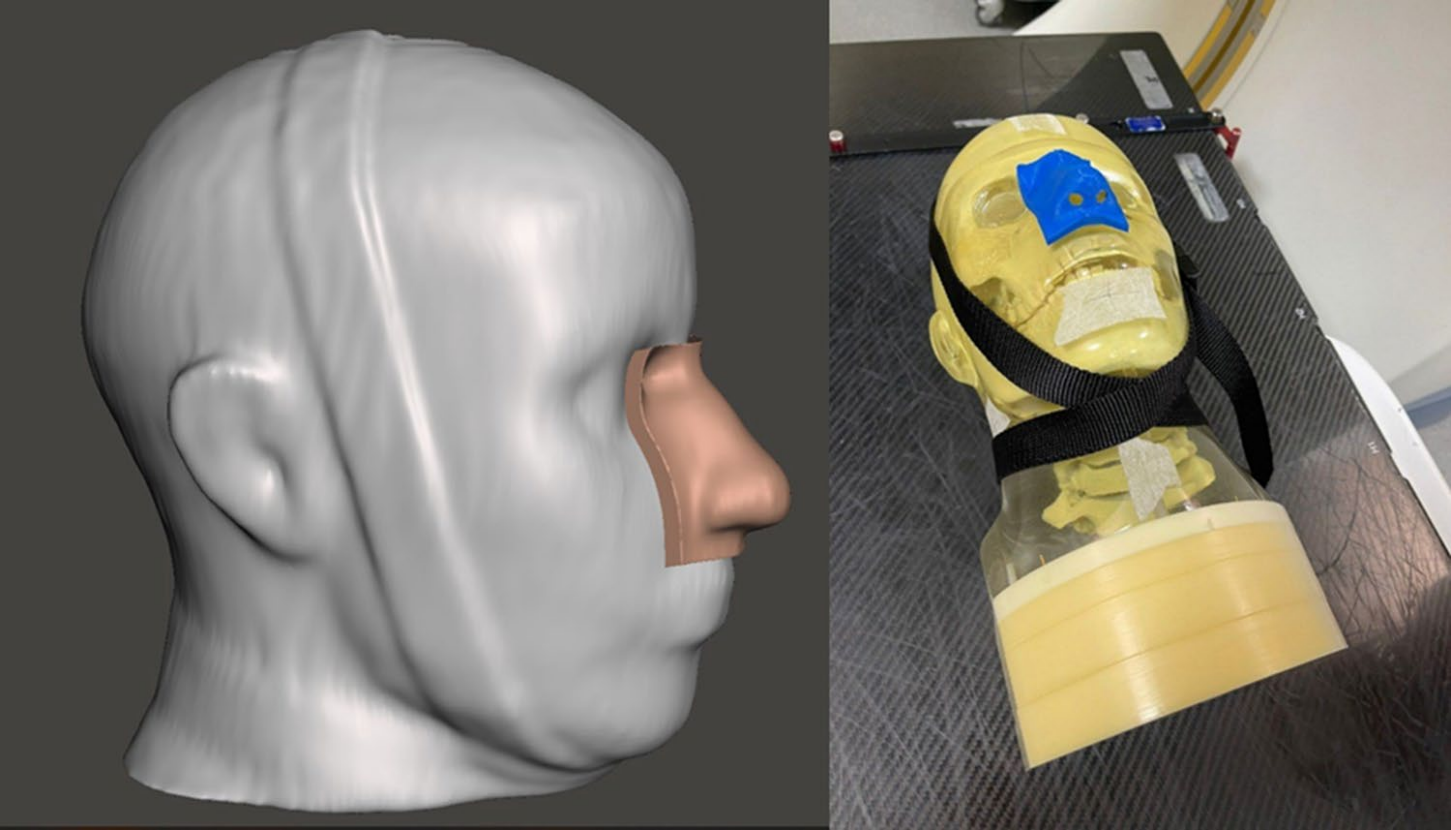
3D model and sample of the bolus to be applied to the nose

Using the aforementioned methodology, the 3D model of the phantom head was obtained, from which the region of the nose was extracted (Fig. 4).

Nose boluses were printed in three different orientations and in triplicates, to study the impact of automatically generated supports and warping due to internal stresses on the fitting of the bolus to the face geometry. Further, the model with the best performance was printed also in TPU to compare the fitting between a rigid material and a compliant material.

Air gaps were assessed by performing a CT scan of each printed sample on the phantom, and segmenting air with Slicer3D software through the shape-based segmentation methodology, obtaining 3D models of air gaps. Air gap volume and the maximum deviation between the bolus and phantom surface were obtained from the Meshmixer software (Autodesk, California, USA) and used to compare nose boluses.

#### Cheek boluses and dosimetric characterization

**Fig. 5.**
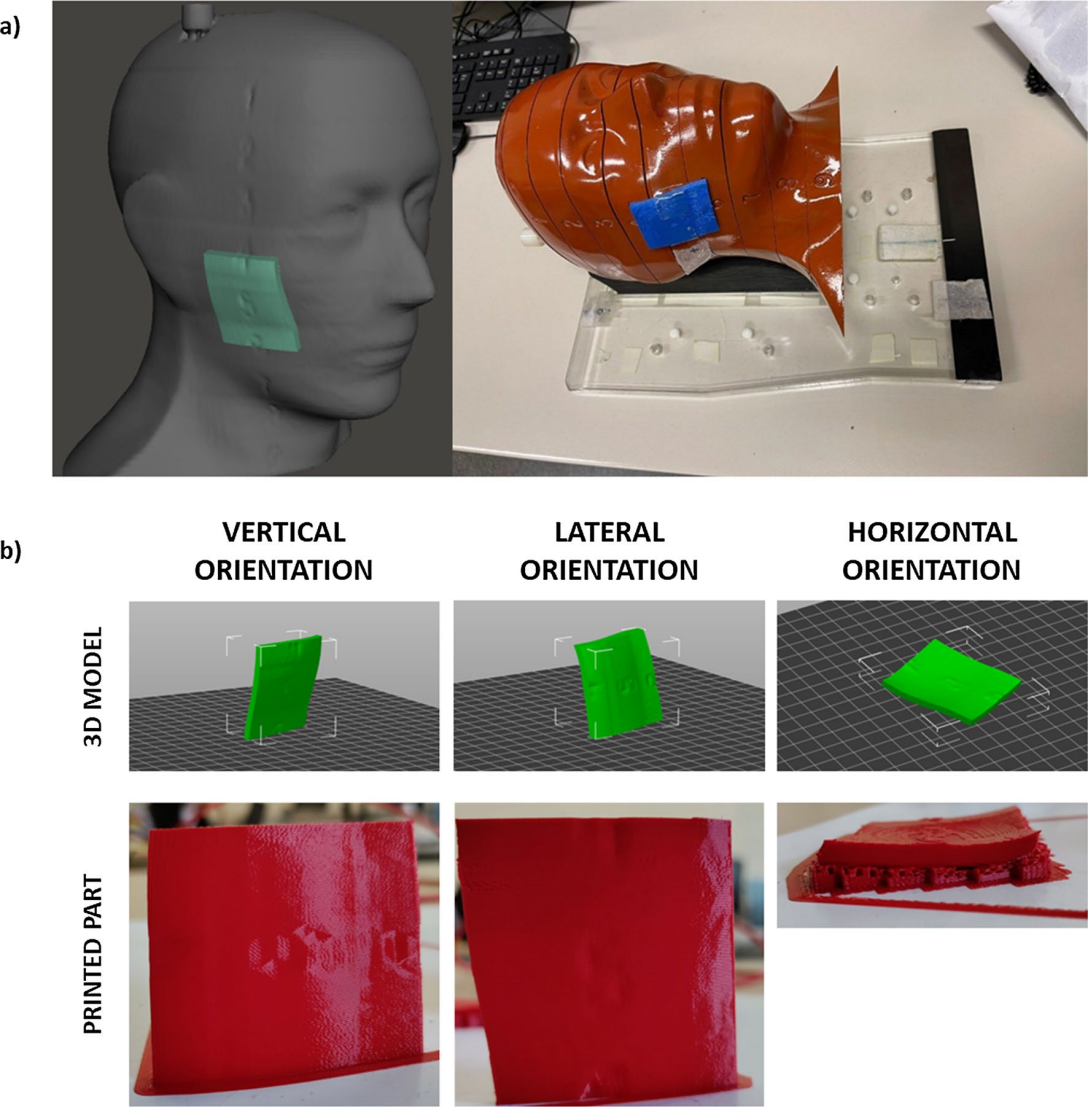
3D model and sample of the bolus to be applied to the cheek

Using the same phantom CT scan and the same infill parameters from the optimization process described in 2.1, cheek boluses (Fig. 5) were printed in PLA in three orientations and in triplicates to evaluate the shift in depth-dose profile.

## Results

### Optimal printing parameters

The depth at which the dose value peaked along with the value of the maximum dose was recorded, and led to the results shown in Table 3.

**Table 3:** Mean dose shifts of the different samples

Pattern type	Infill density [%]	Depth at Dose Max, batch 1 [mm]	Depth at Dose Max, batch 2 [mm]	Mean Dose Shift [mm]	Mean of Depth at Dose Max, of all samples of a given infill type [mm]	Mean of Dose Shift, of all samples of a given infill type[mm]
Grid	40	14,1	12,3	1,30	12,30	−1,80
Grid	50	12,4	12,4	2,10		
Grid	60	12,0	10,6	3,20		
Gyroid	40	11,6	11,0	3,20	10,65	−0,15
Gyroid	50	10,9	10,2	3,95		
Gyroid	60	10,6	9,6	4,40		
Cubic	40	13,0	12,0	2,00	11,82	−1,32
Cubic	50	12,0	10,6	3,20		
Cubic	60	12,4	10,9	2,85		
Rectilinear-10	40	13,1	12,7	1,60	12,30	−1,80
Rectilinear-10	50	12,3	12,7	2,00		
Rectilinear-10	60	11,0	12,0	3,00		
Rectilinear-15	40	13,8	12,0	1,60	12,42	−1,92
Rectilinear-15	50	12,7	10,9	2,70		
Rectilinear-15	60	12,4	12,7	1,95		
Rectilinear-20	40	12,3	12,0	2,35	11,75	−1,25
Rectilinear-20	50	12,7	10,6	2,85		
Rectilinear-20	60	12,0	10,9	3,05		
Conventional	//	10,5		4,00		
No bolus	//	14,5		//		

From this set of results, it can be seen that the conventional bolus shifts the peak of the beam by 4 mm. Taking in consideration the average of all samples per type of infill, it can be noticed that:

- gyroid infill type produced the closest results to the conventional bolus by giving a mean value of 10.65 mm, which is on average 0.15 mm over the benchmark value of the conventional bolus.
- rectilinear infill with 15 degrees of offset between subsequent layers produced the furthest results from the benchmark value of the conventional bolus by giving a mean dose depth of 12.42 mm, which is 1.92 mm under the depth value of the conventional bolus.

**Fig. 6:**
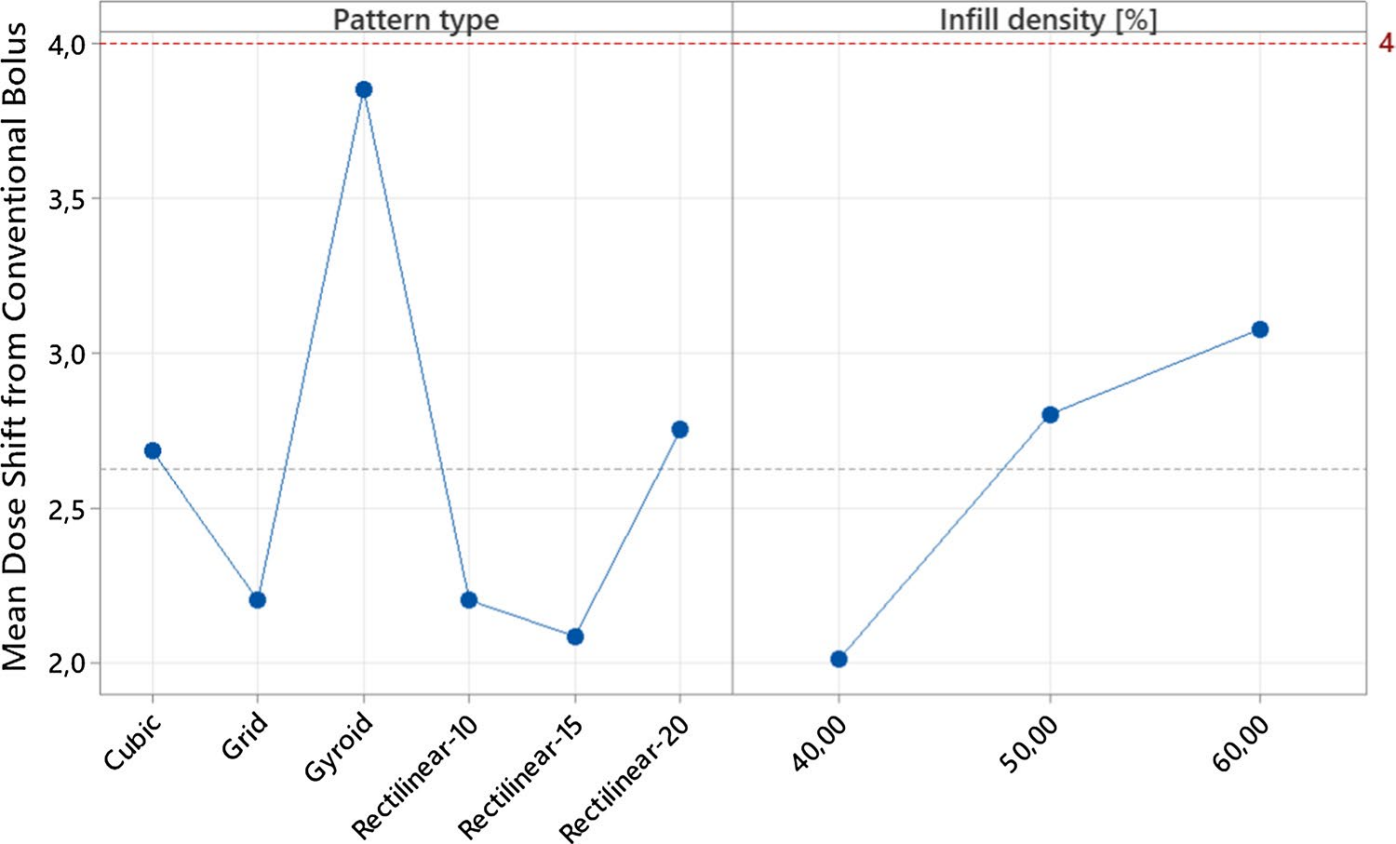
Main effect plots for pattern type and infill density. The left panel shows how the mean dose shift varies for each infill type tested. The right panel shows how the mean dose shift varies for each infill density tested. The dashed red line indicates the conventional bolus performance. The dashed grey line represents just the overall mean response of all the conditions considered

Main effect plots were made for the pattern type and infill density (Fig. 6). The figure shows the mean of the various conditions tested, compared to the overall mean response (grey dashed line) and the conventional bolus response (red dashed line).

From the main effect plots, regarding the infill type, it can be seen the best performing infill is the gyroid and the worst-performing infill is the grid. With regard to infill density, on the other hand, as expected from the literature, an increase in performance was observed as the infill density increased.

Considering these results, the optimal set of parameters was finalized as gyroid infill with 60% infill density.

### Air gap characterization

**Fig. 7:**
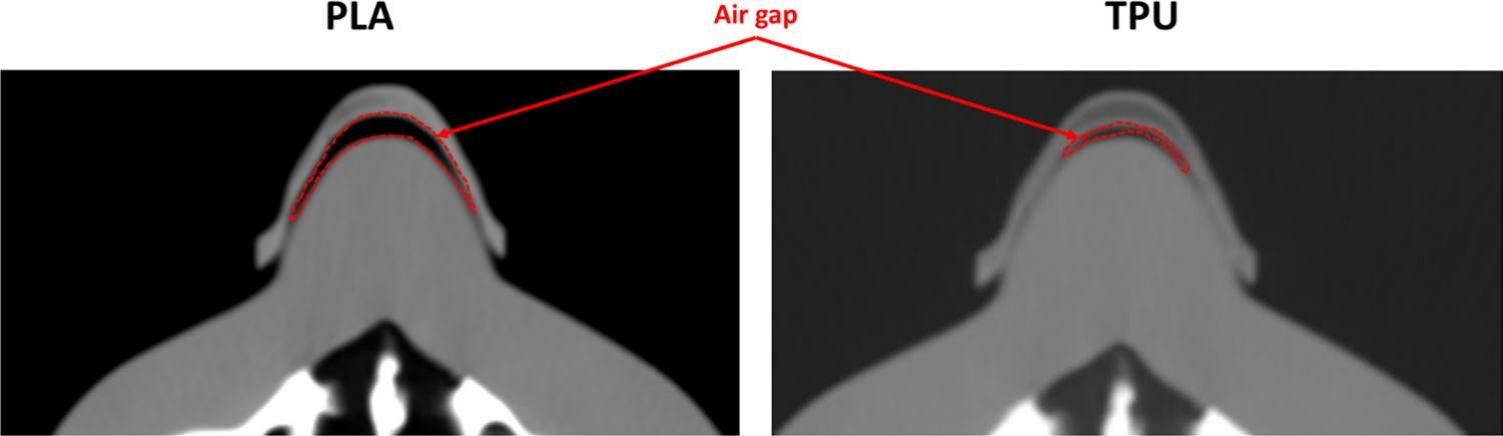
Scan of the phantom with the bolus applied. **a** TPU and **b** PLA

Example of slices from an arbitrary scan are shown in Fig. 7.

**Fig. 8:**
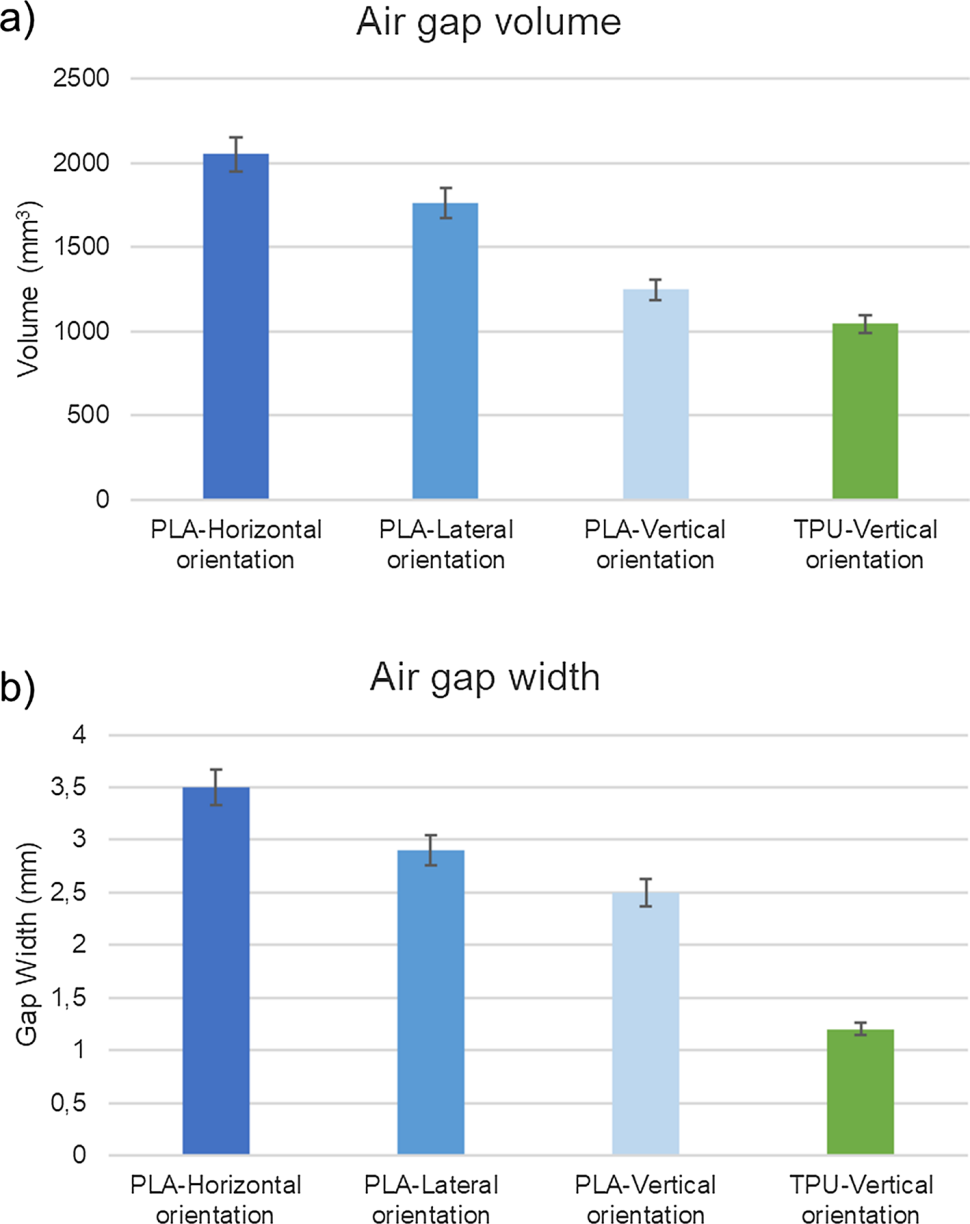
The values of air gap volume **a** and air gap width **b** for the various samples of nose boluses tested are shown in the bar graph. Shaded in blue are the PLA-based samples and in green the TPU-based samples

Based on the results in Fig. 8, it could be seen that within the hard material boluses, the horizontally oriented one performed the worst with the highest air gap volume of 2.05 cm^3^, followed by laterally printed bolus (1.76 cm^3^) and vertically printed bolus (1.25 cm^3^). The vertically printed bolus gave also the least separation value of 2.5 mm, followed by the laterally printed bolus with a value of 2.9 mm. The horizontally printed bolus gave the highest value of 3.5 mm separation between the bolus surface and the skin surface of the phantom.

This led to the vertical print of TPU boluses and consequent testing. The vertically printed bolus with TPU material performed the best in terms of fit to the patient geometry, with an air gap volume of 1.05 cm^3^. The boluses fabricated with PLA have a significant deviation from the result of the TPU bolus. Similarly, the maximum width of the air gap volume follows the same trend, with the TPU bolus having the least deviation from the surface of the phantom, giving a separation of 1.2 mm.

### Dosimetric characterization

**Fig. 9:**
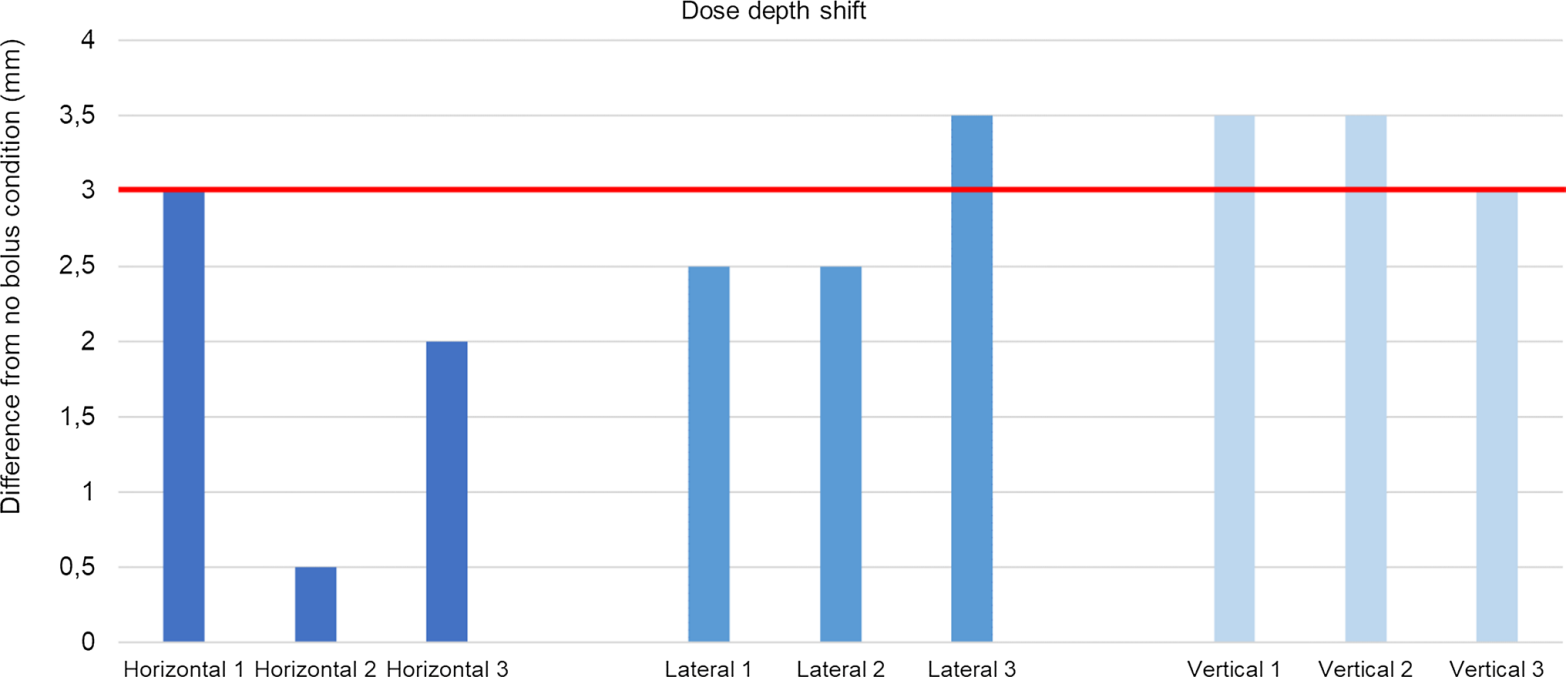
Dose shifts of the three cheek boluses samples of each printing condition

Regarding the cheek boluses, on average, the vertically printed bolus performed better than the boluses printed in the horizontal and the lateral orientations, as shown in Fig. 9 where are displayed the dose shifts of all the samples. The vertically printed boluses gave an average value of dose shift value of 3.33 mm, which is more than the average shift produced by the conventional boluses, equaling a value of 3 mm, the red line in Fig. 9.

The laterally printed boluses gave an average shift of 2.83 mm in the maximum dose position.

The horizontally printed boluses performed the worst of the lot by giving an average shift of 1.83 mm in the position of the maximum dose. Further, there is an outlier shift value of 0.5 mm in the dataset of this specific print orientation. This value indicates that this specific bolus shifted the dose peak by a distance of 0.5 mm alone from the surface of the phantom.

## Discussion

The literature has primarily focused on dosimetric evaluations of 3D-printed boluses with only 100% infill [13–16]. Further, works have been done only on one single type of infill geometry which was primarily rectilinear or grid [11, 15]. This lacuna in research regarding the dosimetric characters in terms of shift in the location of the maximum dose for other available infill geometries, infill percentages and the combinations thereof led the research question of the presented work.

A shift of 3 mm is reported in literature from the peak of the radiation beam without bolus to the peak after the application of 3D-printed bolus [15]. In the cited paper, the bolus is printed with 100% infill. These results in terms of dosimetry are in conjunction with what was obtained from the testing carried out on the designed samples, both in the parallelepipeds and the patient-specific cheek boluses. In general, the shifts observed in the presence of the 3D-printed boluses were comparable to those caused by the conventional boluses.

Regarding the infill characterization of parallelepipeds, the results show that:

- Gyroid performed the best in terms of the dose depth because these infills guaranteed the presence of material in path of the beam wherever the beam might impinge on the bolus. This ensured more interaction between the beam and the material itself and essentially shifted the peak of the beam further backwards. This could not be said for the rectilinear and the grid infills as there was a certain quantity of empty space that is maintained throughout the depth of the bolus. This flaw is illustrated graphically below as screenshots from the slicer software. This result was reported by [17] as well.
- With regards to the infill percentage, more infill percentage essentially meant a denser bolus which in turn ensured the presence of more material to attenuate the beam.

With respect to the air gap evaluations, Fujimoto et al. [15] have studied 3D-printed boluses of the nose area in terms of the presence of air gap and other dosimetric parameters. It was found through the investigation that a commercial bolus produced an air gap of 8 mm which was reduced to 2 mm by a 3D-printed bolus. Dyer et al. [18], have compared the air gap volume and the width of the gap between a Planar Commercial Bolus and a 3D-printed bolus of a similar facial area including nose and adjoining areas. With regards to the width of the air gap, a deviation of 5.69 mm was found between the bolus surface and the skin for Planar Commercial Bolus. According to their study, an air gap beyond 5 mm is said to “negatively impact radiation delivery at the skin surface.” All the cases of 3D-printed bolus, irrespective of being printed from a soft or hard material, have an air gap width of less than 5 mm, ranging from 3.5 mm at the maximum to 1.2 mm at the minimum. This shows that, as an overarching rule, 3D-printed boluses fit the patient geometry better than any commercially available bolus.

As was expected, the TPU bolus performed the best in terms of the fit to the patient profile. This was so due to the inherent compliance that could be observed in the material. The bolus fabricated with this material was elastic and retained its shape despite being subjected to bending stresses.

The intra-orientation trend within the boluses printed with hard material can be explained by the presence of support structures and warpage in the printed samples. Warpage and part distortion, resulting from residual thermal stresses in the part, may be responsible for deviations in the interior surface of the bolus from the originally designed surface`. Warpage is likely to cause a distortion in the part, thereby changing its shape from the original surface that serves as the basis for the bolus structure.

In case of horizontally printed boluses, the support structures of varying heights covered the whole external surface of the bolus, to attach each point of the undulating surface of the nose to the build plate. The other two orientations printed without the need for extensive supports as for the previous case, showed less surface roughness.

Further, the length of the “stacking layers” in the beginning of the print is the shortest in the horizontal orientation (11.33 mm), followed by vertical (43.16 mm) and then lateral (68.44 mm). Based on the works of Wang et al. [19], for a fixed layer number, lower “stacking length” corresponds to higher warp deformation. In case of vertical and lateral orientations, the stacking lengths are almost consistent with the increase in layer numbers. But for the horizontal orientation, the layer length would be the least in the beginning and would increase as the print proceeds. As the layer number increases, the deformation converges for all “stacking layer” lengths, as showed by Wang et al. This trend can also explain the high deviation of 3.5 mm of the internal bolus surface from the surface of the phantom at the nose tip for the horizontally printed bolus, since in this case, the tip of the nose is the portion that corresponds with the lower layer number and the lower “stacking layer” length as the tip is the region that is printed initially for the horizontal bolus.

Similarly, with respect to the dosimetric evaluations of the cheek boluses, the results of Fig. 9 showed that the gyroid infill type performs similar to the conventional bolus material even with an infill percentage of 60%. This shows that even with a reduced material mass for a given volume of bolus, 3D-printed boluses have a possibility to perform better than the conventional bolus material. This observation aims at lesser material usage than a conventional bolus to obtain similar or better dosimetric results.

As can be seen from the results in Fig. 9, a decreasing trend is seen in maximum dose depth as the print orientation changes from vertical to lateral to horizontal. Further, the horizontally printed bolus showed the highest variability, along with the presence of one outlier value. This behavior could be explained by the presence of high surface irregularities on the outer face of the bolus, which are present as remnants of the support structures. Also, a significant amount of “staircasing effect” can be discerned in the horizontally printed bolus which can act as sites for accumulation of air between the bolus and the patient surface. The irregular surface and the inside surface with staircase effect are shown below, alongside the surface finish of other two boluses printed vertically and laterally.

Although landmark points were taken on the phantom for placement of 3D-printed boluses, there could be a discrepancy in placing the bolus structure in the correct position every time. These shifts from the intended position of the bolus may again produce shifts in the dose positions because of high surface irregularities.

Boluses printed vertically and laterally do not require any support structures to be printed because of the absence of any significant overhangs. This lack of support structures indicates the presence of clean surfaces with lesser aberrations which can cause discrepancies in the transmittance of the incident beam.

To summarize, it has been shown in the relevant cited literature that 3D-printed boluses show a dosimetric performance similar to conventional boluses with an additional advantage of conforming to the patient geometry. The identified gap in literature was the study on the effects of print parameters such as infill geometry, infill percentage, and print orientation on the dosimetric characteristics of the 3D-printed boluses. The present work has attempted to address this lacuna through the characterization of various combinations of standard infill geometries and percentages. The clinical evaluation showed that the combination of the gyroid infill geometry and a higher infill percentage of 60% performed similar to a conventional bolus. Utilizing these results, a further characterization was carried out on basis of print orientation on dosimetry and fit to the patient profile on patient-specific boluses. The results of these tests showed that the vertical orientation performs the best, both in terms of shifting the dose peak and fit to the patient geometry. These results show that a 3D-printed bolus does not need to have a solid infill to perform similar to a conventional bolus, and thus an object of reduced weight can be placed on the patient and can be expected to act as required.

As future developments to the work here presented, one could look into dosimetric characterization of flexible materials like TPU-based materials of differing Shore Hardness values. As discussed, TPU-based materials exhibit superior fit than the ones printed with hard material. It would be sensible to quantify the characteristics of the materials in terms of dosimetry and obtain optimal infill parameters as was done for the PLA material that has been reported in this work. Further, a new class of materials specifically designed to print radio-opaque objects can be studied and characterized in terms of dosimetry and fitment. The available materials can also be characterized based on the behavior on extended exposure to X-ray beams on factors such as dimensional changes and material deterioration. Methodology described herein to isolate the region of interest (ROI) and print the bolus can be automated where the physician can merely delineate the ROI and the applicable bolus is modeled, thus reducing human intervention. Within this context, novel scanning methodologies can be explored wherein the patient does not need to undergo superfluous and unnecessary CT scans and the subsequent harmful radiations. Scanning technologies like laser scanning and photogrammetry can be employed to obtain the patient’s geometric information.

Additional AM methodologies can be explored for the fabrication of the boluses as well, which will open up the possibilities to explore a higher range of materials. For example, Vat Polymerization process provides a possibility to study the dosimetry of photosensitive resins, or Selective Laser Sintering provides the same possibility for powder-based materials.

## Conclusions

AM has expanded its applications beyond traditional industries. In healthcare, AM's customizability and rapid prototyping capabilities are harnessed to create patient-specific devices, improving comfort and ensuring a better fit to the patient's body geometry.

In this study, the focus is on 3D-printed boluses and their dosimetric characteristics in comparison to conventional boluses in oncological radiotherapy. The study found that 3D-printed boluses can perform similarly to conventional ones while conforming to the patient's geometry. Factors such as infill geometry, infill percentage, and print orientation were studied, revealing that gyroid infill geometry and a higher infill percentage of 60% yielded results comparable to conventional boluses. Vertical print orientation was identified as the most effective in terms of shifting the dose peak and patient fit.

In contrast to conventional bolus fabrication, which involves complex and error-prone procedures such as heating materials or mixing powders, 3D printing offers a streamlined and cost-effective solution for creating patient-specific boluses in the medical field. By minimizing human intervention and reducing the risk of errors, 3D printing not only saves time but also allows for the integration of low-cost 3D printers in hospitals, enhancing efficiency and repeatability in bolus production. Future developments could include the exploration of dosimetric characteristics of flexible materials like TPU, the study of radio-opaque materials, and the automation of bolus modeling. Novel scanning methodologies and alternative additive manufacturing processes may also be considered for further research.

## Abbreviations

3D: Three-dimensional
AM: Additive manufacturing
CT: Computed tomography
EBRT: External beam radiation therapy
FFF: Fused filament fabrication
IEO: European Institute of Oncology
MU: Monitor units
PLA: Polylactic acid
RGB: Red–green–blue
SSD: Skin-to-source distance
TPU: Thermoplastic polyurethane

## References

[1] Wang X et al. The Clinical Application of 3D-Printed Boluses in Superficial Tumor Radiotherapy. Front Oncol, 2021;11. 10.3389/fonc.2021.698773 10.3389/fonc.2021.698773 PMC8416990 34490095

[2] Dahn HM et al. The use of bolus in postmastectomy radiation therapy for breast cancer: A systematic review. Crit Rev Oncol Hematol 2021; 163. 10.1016/j.critrevonc.2021.103391 10.1016/j.critrevonc.2021.103391 34102286

[3] Kumar L, Haleem A, Tanveer Q, Javaid M, Shuaib M, Kumar V (2017). Rapid Manufacturing: Classification and Recent Development. International Journal of Advanced Engineering Research and Science (IJAERS).

[4] WO2008054823A2 - Bolus materials for radiation therapy and methods of making and using the same - Google Patents. Accessed: Jan. 12, 2023. https://patents.google.com/patent/WO2008054823A2

[5] Lukowiak M (2016). Use of a 3D printer to create a bolus for patients undergoing tele-radiotherapy. International Journal of Radiation Research.

[6] Kang D, Wang B, Peng Y, Liu X, Deng X. Low-cost iPhone-assisted processing to obtain radiotherapy bolus using optical surface reconstruction and 3D-printing. Scientific Reports. 2020;10(1):8016. 10.1038/s41598-020-64967-5 10.1038/s41598-020-64967-5 PMC7228923 32415217

[7] Canters RA (2016). Clinical implementation of 3D printing in the construction of patient specific bolus for electron beam radiotherapy for non-melanoma skin cancer. Radiother Oncol.

[8] Dipasquale G, Poirier A, Sprunger Y, Uiterwijk JW, Miralbell R. Improving 3D-printing of megavoltage X-rays radiotherapy bolus with surface-scanner. Radiation Oncology. 2018;13:1-8.10.1186/s13014-018-1148-1 10.1186/s13014-018-1148-1 PMC6194575 30340612

[9] Ehler ED, Sterling DA (2020). 3D printed copper-plastic composite material for use as a radiotherapy bolus. Physica Med.

[10] Kairn T (2021). Determining tolerance levels for quality assurance of 3D printed bolus for modulated arc radiotherapy of the nose. Phys Eng Sci Med.

[11] Ricotti R (2017). Dosimetric characterization of 3D printed bolus at different infill percentage for external photon beam radiotherapy. Physica Med.

[12] Park SY, Choi CH, Park JM, Chun MS, Han JH, Kim JI (2016). A Patient-Specific Polylactic Acid Bolus Made by a 3D Printer for Breast Cancer Radiation Therapy. PLoS ONE.

[13] Bolus materials for radiation therapy and methods of making and using the same, Nov. 2007

[14] Burleson S, Baker J, Hsia AT, Xu Z (2015). Use of 3D printers to create a patient-specific 3D bolus for external beam therapy. J Appl Clin Med Phys.

[15] Fujimoto K, Shiinoki T, Yuasa Y, Hanazawa H, Shibuya K (2017). Efficacy of patient-specific bolus created using three-dimensional printing technique in photon radiotherapy. Phys Med.

[16] Van der Walt M, Crabtree T, Albantow C (2019). PLA as a suitable 3D printing thermoplastic for use in external beam radiotherapy. Australas Phys Eng Sci Med.

[17] Rao GK, Shah T, Shetty VD, Ravi B. Custom design & fabrication of 3D printed cast for ankle immobilisation. KnE Engineering. 2017 9:98.10.18502/KEG.V2I2.601.

[18] Dyer BA (2020). Characterization and clinical validation of patient-specific three-dimensional printed tissue-equivalent bolus for radiotherapy of head and neck malignancies involving skin. Phys Med.

[19] Wang TM, Xi JT, Jin Y (2007). A model research for prototype warp deformation in the FDM process. Int J Adv Manuf Technol.

